# Experience of Japan in Achieving a Total Ban on Asbestos

**DOI:** 10.3390/ijerph14101261

**Published:** 2017-10-20

**Authors:** Sugio Furuya, Ken Takahashi

**Affiliations:** 1 Japan Occupational Safety and Health Resource Center, Tokyo 1360071, Japan; 2 Asbestos Diseases Research Institute, University of Sydney, Sydney 2139, Australia; ken.takahashi@sydney.edu.au

**Keywords:** asbestos, ban, national experience, international cooperation

## Abstract

This paper aims to examine the process through which a total ban on asbestos was achieved in Japan. We reconstructed the process, analyzed the roles of involved parties/events, and drew lessons from the Japanese experience of achieving the ban. In Japan, a bill to phase out asbestos was proposed in 1992 but rejected without deliberation. Wide support for such a ban subsequently grew, however, largely due to the actions of trade unions and civil societies in establishing a coalition, raising awareness, organizing asbestos victims and their families, and propagating information on international developments. A governmental decision towards a ban was made in 2002 based on several national and international factors. A huge asbestos scandal in 2005 preponed the achievement of a total ban and led to the establishment of comprehensive measures to tackle asbestos issues. However, challenges remain for the elimination of asbestos-related diseases.

## 1. Introduction

Japan has the world’s 10th-largest population, with over 127 million people. In 2016, Japan had the third largest national economy in the world in terms of national GDP.

**Figure 1:**
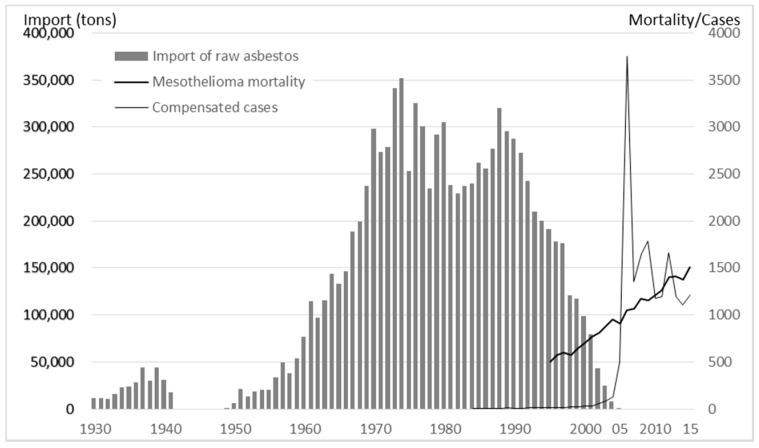
Import of raw asbestos, mesothelioma mortality, and number of mesothelioma cases compensated by Workers’ Compensation Insurance (since 1978 when the first case was compensated) and the Asbestos Victims Relief schemes (since 2006) in Japan.

Almost all asbestos used in Japan was imported. Raw asbestos was not imported during World War II (WWII), but imports increased again thereafter to attain a peak of 352,110 tons in 1974. Asbestos imports began consistently decreasing in 1989 and have been at zero since 2006. The amount of raw asbestos imported during the period of 1930–2005 totaled 9,879,865 tons (Figure 1) [1].

Data on mesothelioma mortality in Japan has been captured in the vital statistics since 1995. Five hundred mesothelioma deaths were recorded in 1995, and this number steadily increased to reach 1504 in 2015. In 1987, the first mesothelioma case was recognized as an occupational disease and compensated by Workers’ Compensation Insurance. The cumulative number of compensated mesothelioma cases was around 500 during the 18-year period of 1987–2004; in contrast, 500 such cases were recorded in 2005, and 1000 were recorded in 2006. In addition, the Asbestos Victims Relief Act was enacted in 2006 to compensate asbestos-related disease (ARD) victims not covered by the workers’ compensation schemes (Figure 1) [1].

This paper aims to examine the process through which a total ban on asbestos was achieved in Japan.

## 2. Materials and Methods

The authors referred to the relevant documents and the experience of the first author (S.F.) as the coordinator of the Ban Asbestos Network Japan (BANJAN) since 1996. From these sources, we reconstructed the process, analyzed the roles of involved parties/events, and drew lessons from the Japanese experience.

## 3. Results

### 3.1. Initial Regulations on Asbestos Use

In Japan, regulations against asbestos hazards began with measures to protect workers’ health from asbestos as a causative substance of asbestosis. Prior to WWII, asbestos was recognized as causing asbestosis. The Pneumoconiosis Act, which was enacted in Japan in 1960, aimed to prevent asbestosis and manage the health of asbestos-exposed workers.

In 1975, the Ordinance on Prevention of Hazards Due to Specified Chemical Substances was amended to introduce stricter measures to prevent occupational cancers due to asbestos exposure. Concurrently, the spraying of asbestos was recognized as high-risk work and prohibited in principle, and the Ministry of Labor (MOL) issued an administrative notice encouraging that substitutes be used in place of asbestos materials, but with priority on crocidolite. However, this administrative guidance and the voluntary efforts of industries were insufficient to totally end the use of asbestos. Indeed, asbestos imports actually increased again in the late 1980s (Figure 1).

Regarding public health and the environment, measures to protect both against asbestos were introduced by the Air Pollution Control Act in 1989 and the Waste Disposal and Public Cleaning Act in 1992.

### 3.2. Call for a Total Ban on Asbestos: The Role of BANJAN as an NGO

The call for a total ban on asbestos was first raised in the late 1980s. Following the adoption of ILO Convention No.162 on Asbestos, BANJAN was established in 1987 as a coalition of: (i) trade unions; (ii) groups representing consumers, environmental protection, occupational safety and health (OSH), and other areas; and (iii) individuals from various other fields. The participants believed that such a coalition was critically needed to tackle various asbestos-related issues by raising awareness that asbestos is not only an occupational hazard, but also a public health and environment hazard. Top priority was given to achieving a total ban on asbestos in Japan as early as possible [2]. BANJAN, in cooperation with professionals and politicians, drew up a bill to phase out asbestos and asbestos-containing materials (ACMs). The opposition parties presented the bill to the national diet in 1992, but the ruling party rejected it without deliberation.

The issue of “asbestos in school” was widely publicized in 1986/87, but the attention of the media and general public quickly waned. Around that time, several industries moved to using substitutes for asbestos: for example, the automobile industry stopped using asbestos for new products by 1996. However, the Japan Asbestos Association (JAA), which was established by asbestos manufacturers/importers and their associations in 1948, strongly opposed the 1992 bill and lobbied politicians to that effect. BANJAN held meetings with the JAA but could not reach any agreement. Some trade unions from asbestos-manufacturing companies also opposed the bill. Importantly, asbestos-related diseases (ARDs) were still not well known to the public at this time; data on mesothelioma mortality did not become available through the vital statistics until 1995.

In Japan, the Occupational Safety and Health Act (OSHA) gives the government the authority to prohibit the manufacture, import, transportation, supply, and use of a substance that is identified in its Enforcement Order and can seriously impair the health of workers (article 55). In 1995, the government amended the OSHA Enforcement Order to prohibit the use of crocidolite, amosite, and materials containing more than 5% of either by weight. According to JAA, the asbestos industry in Japan had voluntarily stopped using crocidolite and amosite by 1987 and 1992, respectively [3].

In the meantime, BANJAN expanded its efforts to organize actions, such as awareness-raising events and annual meetings with officials of the relevant ministries, as well as to provide consultation services and support for asbestos victims and their families, and to offer relevant information (particularly regarding the latest international developments) to the media, policy makers, and the general public. BANJAN also supported the actions of various organizations/individuals with respect to asbestos issues. Together, these grass-roots efforts prompted gradual changes in society at large.

### 3.3. Governmental Decision towards a Total Ban

The most important asbestos-related governmental decision was made in 2002. On 28 June, the Minister of Health, Labor and Welfare (MHLW) announced his intention to introduce an in-principle ban on chrysotile asbestos [4]. This was a significant change from the “controlled use” policy long held in Japan. The authors believe that the following factors triggered this policy shift:

(1) On 2 April 2002, at the 75th Meeting of the Japan Society for Occupational Health (JSOH), researchers presented a scientific paper on estimating the future mortality from malignant pleural mesothelioma in Japan. The full English-language paper was published in 2005 [5]. The study was covered on 2 April by a major national newspaper (Asahi Shimbun), with the headline “Asbestos Deaths Likely to Soar”. Other media outlets soon followed. On 17 April, BANJAN held an emergency meeting to address the study. At the meeting, the widows of two deceased mesothelioma victims described their fights against the deadly disease to the public for the first time.

(2) On 20 May 2002, more than 10 asbestos-related disease victims and their families held a meeting with officials from the MHLW. These individuals made the first call for an immediate total ban on asbestos, with the goal of avoiding unnecessary deaths in the future. The media coverage reported the officials’ response as “lukewarm”.

The Minister’s announcement on 28 June 2002 came directly after these events. In the authors’ opinion, the increasing visibility and voices of the asbestos victims were the most important factors in spurring Japan to move towards a total ban on asbestos. It should be noted that, in 2002, there were 810 mesothelioma deaths (vital statistics), but only 55 cases were compensated. The small proportion of compensated cases reflected that, although the burden of ARDs became increasingly visible, the awareness of the general public, including the understanding that ARDs are compensable, was still low. The aforementioned call by the victims thus played an important role to push the societal momentum towards an asbestos ban.

(3) In 1998, Canada lodged a complaint with the World Trade Organization (WTO) against the previous year’s French ban on asbestos, claiming that it violated free-trade principles. Asbestos thus became a subject of a so-called “international trade dispute”. Without waiting for the conclusion of this dispute, the European Commission adopted Directive 1999/77/EC to prohibit asbestos beginning 1 January 2005. The WTO made its final decision in 2001: It rejected the Canadian complaint [6] and concluded that “it is undisputed that WTO Members have the right to determine the level of protection of health that they consider appropriate in a given situation”. The WTO response also agreed that the (French) measure “protects human life or health” and “no reasonably available alternative measure” existed, and upheld the finding that the ban was justified. Although this was not extensively covered by the Japanese media, several players were influenced by this outcome; this included BANJAN, which made efforts to inform these global developments to the public.

(4) The MHLW convened an inter-ministerial meeting on 29 March 2002, inviting representatives from five other ministries to review their policies on asbestos. The ministries had varied levels of awareness and concern regarding asbestos, and the meeting failed to address a ban on asbestos (probably due to the lack of political leadership). Once the MHLW made its decision, however, it was not opposed by any other ministry. After the “Kubota Shock” (see Section 3.7 for additional details), the MHLW referred the amendment of the International Convention for the Safety of Life at Sea (SOLAS) by the International Maritime Organization (IMO) to prohibit new installation of ACMs on all ships as of 1 July 2002 [7]. The MHLW’s decision is also likely to have been impacted by the International Agency for Research on Cancer (IARC)’s 2001 decision not to classify glass wool, rock wool, etc., as carcinogens [8].

(5) BANJAN held its first direct dialog with the JAA on 9 February 2001. The JAA maintained that the “controlled use of asbestos” was safe and stated that the association had never discussed the possibility of ceasing the use of asbestos. However, the WTO’s decision (see above) influenced some major manufacturers to stop using asbestos around this time. Indeed, at the next meeting between BANJAN and the JAA, which was held on 5 September 2002, the JAA admitted that a ban on chrysotile would depend on a political decision or a market shift.

(6) In 2001, Japan’s largest trade union national center, JTUC-RENGO established its position to support a total ban on asbestos. Also in 2001, the JSOH set the reference values for occupational exposure at 0.15 f/mL for chrysotile and 0.03 f/mL for non-chrysotile asbestos, which corresponded to 10^−3^ individual excess lifetime risks of mesothelioma and lung cancer.

(7) In 2002, the Asbestos Symposium for Asian Countries was co-organized in Kitakyushu by the University of Occupational and Environmental Health, Japan and the Finnish Institute of Occupational Health.

### 3.4. Total Ban on Asbestos in 2004: The “Negative List”

After the Minister’s announcement, the MHLW conducted a “Survey on the Substitution of Asbestos and ACMs” among manufacturers, asbestos users and their organizations in order to identify the uses of asbestos for which substitution was difficult at the time, and the expected timing of substitutions becoming possible. The results of the survey were released on 12 December 2002 [9]. At this point, the MHLW set up its “Committee on the Substitution of Asbestos” to review the survey results and recommend possible regulations [10]; the committee published its report on 4 April 2003 [11].

In this report, the committee identified 197 asbestos-containing products that were at the time on the market, grouped them into 10 product categories, and examined the possibility of substitution for each product category. The committee concluded that substitution would be possible for all five categories of construction products (extruded cement panels, decorated cement shingles for dwelling roofs, fiber-reinforced cement boards, fiber-reinforced cement siding, and asbestos cement pipes) and two of the five categories of non-construction products (adhesives for insulators and friction materials for brakes and clutches). Regarding the remaining three non-construction products (heatproof/electric insulation board, joint sheets, and sealing material) the committee found that “some of those products can be replaced by non-asbestos materials, but others cannot be substituted from the viewpoint of security” and that “it is presently difficult to specify such products as substitutable (or not) with respect to specific conditions, such as the temperature, the type of equipment for which the product is being used, etc.”. Also, the committee reported that “asbestos cloth and yarn are currently used as sealing materials, so the substitution of those products will depend on the substitution of sealing materials.”

The MHLW proposed that the OSHA Enforcement Order be revised to prohibit products of the seven substitutable categories (see above) that contained asbestos at more than 1% by weight (a reduction from 5%). In this way, the MHLW took a “negative list” approach by listing only prohibited products. The proposal was put forth on 2 May 2003, and was followed by a public consultation [12]. At this consultation, BANJAN advanced a counterproposal that called on the MHLW to: (i) adopt a “positive list” approach; (ii) cover ACMs containing asbestos at more than 0.1% by weight; (iii) achieve a total ban by 2005 at the latest; and (iv) prevent the overseas transfer of ACM manufacturing. Of about 90 respondents, only 9 opposed the prohibitions proposed.

The Japanese Government notified the WTO of its plan to prohibit those ACMs [13]. The Asbestos Institute sent a delegation to Japan and a hearing was conducted by the MHLW. Officials from the Embassy of Canada in Japan and the Japan Office of the Quebec Government joined forces to oppose the plan. The MHLW inquired as to the situation of ARDs in Canada; these groups promised to provide information at a later date, but this never happened. Asbestos industries from several other countries also sent letters opposing the Japanese plan. Despite this opposition, the MHLW remained steadfast in its plan to prohibit ACMs, presumably because it was not convinced of the advanced arguments.

The MHLW published the revised Enforcement Order without modification on 16 October 2003. It did not include any target time for achieving a total ban.

### 3.5. Year 2004 as a Turning Point

With respect to the tackling of asbestos-related issues in Japan, the year 2004 can be considered a terminus ad quem of past actions and a terminus a quo for the next stage of efforts. The year 2004 can be marked as such for the following reasons:

(1) After two years of preparation, the Japan Association of Asbestos-Related Disease Victims and Their Families was established on 7 February 2004. Its establishment was fully supported by BANJAN, and the association remains a core affiliate of BANJAN today. This national network of victims/families started with 3 branches and 60 members and has grown to encompass 20 branches and 900 members. At the inception of this group, most of the members were victims due to occupational asbestos exposure.

(2) The revised OSHA Enforcement Order went into force on 1 October 2004. The MHLW called this “a total ban in principle”. The MHLW also reviewed all relevant ordinances and regulations to adapt to post-ban needs, and decided to newly enact the Ordinance on Prevention of Hazards due to Asbestos and ratify ILO Convention No.162 on Asbestos (in 2005).

(3) In 2004, the Global Asbestos Congress 2004 (GAC2004) was held in Tokyo on 19–21 November; it was organized by BANJAN and various other organizations. The MHLW and Ministry of the Environment (MOE) supported GAC2004 [14]. This was the second Global Asbestos Congress, following GAC2000 in Brazil. The Collegium Ramazzini stated that “the declaration (adopted by GAC2004) serves as a beacon to nations around the world” [15]. Since then, BANJAN and its affiliates have been actively involved in the global campaign to ban asbestos.

(4) In the following year of 2005, BANJAN and the newly established national victims’ network met victims of environmental asbestos near an asbestos plant in Amagasaki City, leading to the huge asbestos scandal that became widely known as the “Kubota Shock”.

### 3.6. “Kubota Shock” and “Comprehensive Measures” in 2005

On 29 June 2005, a major national newspaper (Mainichi Shimbun) carried an article disclosing that ARDs had taken the lives of many workers who had labored at a former Kanzaki plant of the Kubota Corporation (a major machinery maker in Japan) in Amagasaki City. This plant had manufactured asbestos cement pipes from 1954 to 1975, and asbestos cement housing materials (mainly roofing and outer wall components) from 1960 to 2001. The article additionally conveyed that five residents who lived or had lived within one kilometer of the plant had been inflicted with mesothelioma, two of whom had already died. None of these victims had a history of occupational asbestos exposure. It could thus be assumed that neighborhood exposure to asbestos leaking from the plant was the cause. From this, the public perception grew that essential information on the health impacts of asbestos had long been concealed.

This huge scandal in Japan was called the “Kubota Shock”. All media followed this story, and various aspects of asbestos issues were intensively covered on a daily basis for more than a year. The relevant ministries were forced to disclose all information, and they requested that a wide range of private and public organizations also verify and disclose relevant information, such as their past use of asbestos, any asbestos victims at their companies, and the existence of ACMs in their buildings and facilities. The MHLW subsequently disclosed the names of all companies associated with ARD cases that had been compensated by Workers’ Compensation Insurance; in the years since, such disclosure has been performed annually [16].

An inter-ministerial meeting of section chief-level officials was held on 1 July 2005; this was upgraded to the department director level on 21 July, and finally to the ministerial level on 28 July. Five such ministers’ meetings were held by the end of 2005; the ministers drew up an “Immediate Measures to Tackle Asbestos Issues” at the first meeting, and revised these measures twice during subsequent meetings. They then established the “Comprehensive Measures to Tackle Asbestos Issues” at the fifth meeting, which was held on 27 December 2005 [17]. The outline of the measures was as follows:

● Relief for all asbestos victims without omission
○ Establishment of a new relief act for asbestos victims not covered by workers’ compensation schemes
○ Publicity to inform public about the workers’ compensation scheme
○ Promotion of studies that contribute to relief of victims
● Proactive measures to prevent further suffering
○ Removal of asbestos from existing facilities
○ Prevention of emission and exposure during demolition of buildings
○ Appropriate disposal of asbestos waste
○ Immediate total ban on asbestos
● Relieving anxieties of the public, including measures for persons exposed to asbestos in the past
○ Identification of the real situation and active information service for the public
○ Health consultation, etc.

Based on these comprehensive measures, a new “Asbestos Victims Relief Act” was enacted in 2006. In addition, the Air Pollution Control Act, the Local Government Finance Law, the Building Standards Act, and the Waste Disposal Act were amended in the same year. As a direct consequence, the number of ARD cases compensated by the Workers’ Compensation Insurance dramatically increased. In addition, compensation began by the new Asbestos Victims Relief schemes. Kubota officially apologized to environmental victims near its plant and their families, and set up a compensation scheme for them. So far, more than 300 cases have applied for such compensation [18].

### 3.7. Total Ban on Asbestos in 2006: A “Positive List”

In relation to the ban, the Minister of Health, Labor and Welfare announced his intention to achieve a total ban on asbestos by 2008, and the Comprehensive Measures stated that the MHLW would prepone this and take necessary measures by the end of 2006. Accordingly, the MHLW set up the “Committee on the Substitution of Asbestos for a Total Ban on ACMs” on 25 August 2005. The report of this committee, which was published on 18 January 2006 [19], recommended that all ACMs be prohibited with the exceptions of asbestos-containing gaskets and specific applications of existing chemical, iron-making, and non-ferrous metal-making plants, where asbestos was considered to be needed as packing for interfaces subject to certain conditions (i.e., very high temperature or pressure). This represented a change from a “negative list” to a “positive list”, and MHLW called it a “total ban on asbestos”.

Accordingly, the OSHA Enforcement Order was amended on 2 August 2006. This amendment, which began being enforced on 1 September 2006, expanded the order to cover materials containing asbestos at more than 0.1% by weight (a reduction from 1%).

### 3.8. A True “Total Ban” Was Achieved in 2012

Acting quickly, the MHLW set up the “Committee on the Substitution of Asbestos for the Derogated ACMs for a Total Ban on ACMs” on 1 September 2006. In its report, published on 28 April 2008 [20], the committee recommended that the derogated ACMs be prohibited in principle by the end of 2008, and noted when the substitution of asbestos was expected for to become possible in each case.

The OSHA Enforcement Order was amended to remove derogated ACMs from the prohibition in 2008, 2009, 2011, and 2012. A literal “total ban” on asbestos, without derogation, was achieved on 1 March 2012 [1].

Notably, however, several challenges still remain in Japan’s efforts to fully implement a “total ban” on asbestos.

(1) The legal ban does not apply to the existing ACMs in buildings and other infrastructures. To ultimately achieve an asbestos-free environment/society, all existing ACMs must be safely removed and disposed of.

(2) Some cases of illegal import and/or use of ACMs have been reported, and the relevant checking system is still insufficient. Of particular concern is the naturally occurring contamination of minerals by asbestos. This applies to both imports from foreign countries and in-country deposit sources.

(3) In Japan, construction waste is often reprocessed and sold as “recycled crushed stones”. As the relevant checking system is far from adequate, these recycled crushed stones must be considered to contain asbestos until proven otherwise. Long-term measures to monitor and remedy contaminated soils/areas and waste disposal sites are lacking.

(4) The legal ban applies to the manufacturing, import, transport, supply, and use of asbestos and ACMs, but does not apply to export.

(5) The translocation of asbestos-related businesses to other countries constitutes a problem that warrants further investigation.

### 3.9. Past Measures Are Now Being Judicially Examined

After the “Kubota Shock”, the relevant ministries committed to verifying the ministerial measures taken in the past and reported back during the above-discussed meetings. The overarching theme of these self-reports was that the past actions of each ministry were generally appropriate. Today, however, some of these past measures are being contested in court.

On 9 October 2014, in addressing a case of asbestos textile plants and local exhaust ventilation systems, the Supreme Court of Japan ruled that the Japanese Government illegally failed to protect workers from asbestos exposure. The MOL obliged employers to install a local exhaust ventilation system at asbestos plants in 1971. However, the ruling stated that the government’s introduction of the relevant measure was too late in light of the purpose and nature of its deputed authority (the Supreme Court ruled that the MOL should have done so in 1958 at the latest). The ruling then judged this as illegal under the State Redress Act, and ordered the Japanese Government to compensate the relevant asbestos victims and their families [21].

To date, six district court decisions have been made regarding cases of ARDs in construction workers, five of which cited government responsibility in the failure to administer measures, such as protective masks. One ruling cited government responsibility in the lack of a full ban on asbestos, stating that the MOL should have prohibited chrysotile asbestos in 1995 when it prohibited crocidolite and amosite. More court rulings are expected in due course.

A lesson can be drawn for the governments of all countries currently using asbestos: whether or not a ban is planned, the government should thoroughly review and consider their current actions against asbestos in view of the historical and current developments in Japan.

## 4. Discussion

Clearly, Japan missed earlier opportunities to adopt a total ban on asbestos by waiting until ARD victims became visible and disputes regarding international trade were concluded. Unfortunately, the ARD epidemic that the country faces today and will continue to experience in the future is a natural consequence of the failure to take early appropriate action. It is clear that the voices of asbestos victims and their families can create a most powerful impetus, as can building coalitions and expanding support for a ban among various social parties (e.g., victims/families, workers, citizens, professionals, politicians, etc.). Media attention is helpful and often powerful, but it is short-lived. Above all, political advocacy for immediate decision making is key.

The Comprehensive Measures established by the ministers’ meetings in 2005, which included a policy for achieving a total ban on asbestos, represented a significant political turning point. However, these measures have never been reviewed or revised. Moreover, it was the “Kubota Shock”, not the introduction of a ban, that improved ARD compensation. It should be emphasized that a ban on asbestos is an important move, but it is only a first step in tackling the wide-ranging legacy of asbestos. Key points for other countries keen to learn from the Japanese lesson are to: (i) secure implementation of a total ban as early as possible; (ii) promote justice for all asbestos victims/families and exposed persons; (iii) aspire to achieve an asbestos-free society/environment for the elimination of ARDs; and (iv) collaborate globally and learn from the experiences of other countries.

## 5. Conclusions

Countries that have not yet introduced a ban on asbestos should adopt a precautionary approach by introducing a total ban on asbestos without waiting for an epidemic of ARDs to appear. There is enormous value in learning from Japan’s example, particularly with respect to detecting and empowering asbestos victims and their families, building coalitions, and implementing comprehensive strategies to eliminate ARDs. Moreover, international instruments that support and facilitate the banning of asbestos are becoming increasingly available [22]. More powerful regional and/or international instruments, such as those directly requiring a total ban on asbestos, are desired. However, the lack of such a push should not be used as an excuse to avoid introducing a national ban right now. The National Program for the Elimination of Asbestos-Related Diseases (NPEAD) [23] can help facilitate the establishment of comprehensive strategies, both for countries that have not yet introduced a ban and for those that have already introduced a total ban on asbestos.

If ARD victims are not yet visible in a country, it does not mean that no real victims exist. Efforts to detect and empower asbestos victims are recommended for all countries. Scientific means to estimate the burden of ARDs (for the world and each country) has substantially advanced in recent years [24]; such estimates are now available through databases such as the “GBD Compare” database [25] and others [26]. Although they are not perfect, these estimates can be used as a basis for establishing policy towards a total ban on asbestos, and for discussing the issue of under diagnosis and how to improve the situation.

Naysayers can continue their so-called “scientific debates” endlessly, so it is very important that policy makers not get tripped up by such arguments. From the Japanese experience, we see that it is more important to highlight, expand, and strengthen positive aspects towards a total ban on asbestos.
